# Anthracene coupled adenine for the selective sensing of copper ions

**DOI:** 10.3762/bjoc.6.44

**Published:** 2010-05-05

**Authors:** Kumaresh Ghosh, Tanushree Sen

**Affiliations:** 1 Department of Chemistry, University of Kalyani, Kalyani-741235, India

**Keywords:** adenine-based anthracene, copper ion selective, fluorescence, metal ion recognition, PET sensory systems

## Abstract

Anthracene-based adenines **1** and **2** have been designed and synthesized, and their metal ion recognition properties have been established fluorometrically. Both molecules exhibit Cu^2+^ induced ON-OFF type signaling patterns over the other representative metal ions studied. Compound **1** exhibits 97% quenching of emission in the presence of Cu^2+^ whilst derivative **2** shows 81% quenching under similar experimental conditions.

## Introduction

**Scheme 1:**
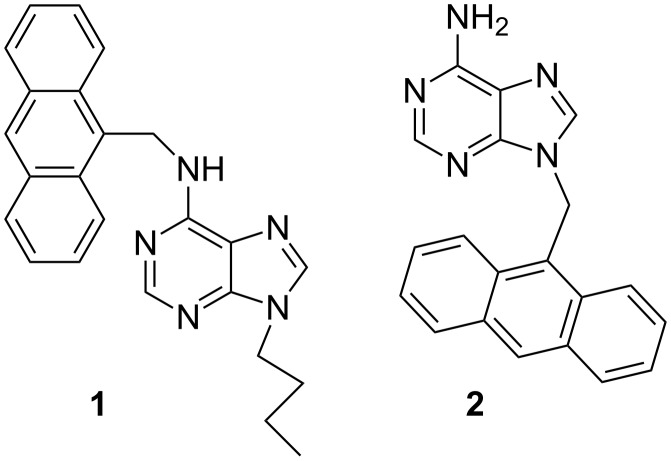
Adenine-based receptors **1** and **2**.

Fluorescent chemosensors for the detection of biologically relevant metal ions have been widely exploited in the field of supramolecular chemistry [1–4]. Among the various transition metal ions, the copper ion draws significant attention due to its crucial role in biological systems. This metal ion causes significant environmental pollution and also serves as a catalytic cofactor for a variety of metalloenzymes [5–9]. However, exposure to high levels of copper, even for a short period of time, can cause gastrointestinal disturbance, while long-term exposure can lead to liver or kidney damage [10–16]. For these reasons, the past few years have witnessed a number of reports on the design and synthesis of fluorescent sensors for the detection of Cu^2+^ ions [17–24]. Although there are various reports in this regard, synthetic receptors with improved binding efficiency are still in demand. In addition, the coordination of the metal ion with nucleobases plays an important role in the stability of the nucleic acid structure [25]. Among the nucleobases adenine provides five interactional sites for coordination with metal atoms. Although it has already been established that adenine moiety exhibits preferential coordination of silver ions [26], the formation of Cu(II) complexes of substituted adenines is also known in the literature [27]. Depending on the smaller size of the substituent at N9 position of adenine, a preferential coordination of Cu^2+^ ion at N7 over N1 or N3 is found to occur for bisadenine ligands [27]. Smaller substituents lead to simultaneous binding of silver ion at all the three centers. However, in this paper we wish to report that introduction of an anthracenyl group (a flat hydrophobic surface) at the different positions in adenine leads to the preferential binding of Cu^2+^ ion over Ag^+^ and other transition metal ions studied. In this connection, adenine-based receptors **1** and **2** (Scheme 1) have been synthesized and their metal ion binding properties have been studied by UV–vis and fluorescence methods. Both **1** and **2** are found to be selective for Cu^2+^ in CH_3_CN containing 0.02% DMSO. Importantly, **1** is found to be more efficient binder than **2** as reflected in the study.

## Results and Discussion

**Scheme 2:**
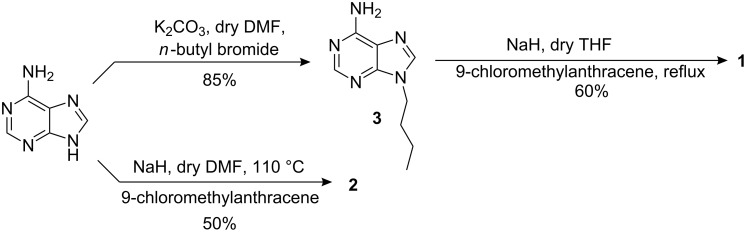
Syntheses of receptors **1** and **2**.

The syntheses of **1** and **2** are outlined in Scheme 2. First, 9-position of adenine was alkylated in the presence of NaH in dry DMF using 9-chloromethylanthracene, obtained from the reaction of 9-anthracenyl alcohol with thionyl chloride, to afford **2** [28] in 50% yield. In a similar manner, 9-butyladenine **3** was obtained from the reaction of adenine with *n*-butyl bromide in the presence of K_2_CO_3_ in dry DMF. Further reaction of **3** with 9-chloromethylanthracene led to the compound **1** [29] in 60% yield. All the compounds were characterized by conventional methods [30].

**Figure 1:**
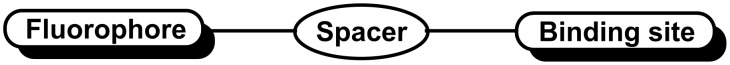
Design principle for **1** and **2**.

The adenine-based molecules **1** and **2** were designed according to the design principle of a PET sensor, shown in Figure 1. In both **1** and **2**, adenine is defined as the binding site, which is connected to the anthracene probe *via* –CH_2_– spacer at the different regions of adenine.

**Figure 2:**
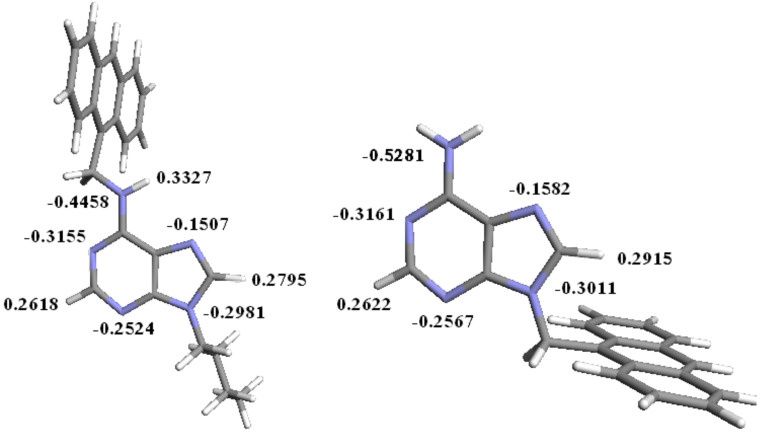
AM1 optimized geometries of **1** (E = −162.48 au) of **2** (E = −139.59 au).

In order to find out the possible site for binding of metal ions, we optimized the geometries of both **1** and **2** at AM1 level in the gas phase [31]. In **1** the disposition of anthracene at the Watson–Crick (WC) site is favored over its orientation at the Hoogsteen (HG) site. This is in accord with the previous report by Engel et al. [32]. We also noted this phenomenon in our previous report [30]. The charge densities at the different nitrogen centers are shown in Figure 2. It is evident from Figure 2, that the WC sites in **1** and **2** provide relatively greater charge density than the HG sites. Thus it is presumed that the binding of metal ion will preferably take place in solution at the WC site in both cases, although the binding at the HG site cannot be ruled out. This is in accord with the observation of Glass et al. [33]. It is worth noting that the introduction of the alkyl group on the amino group of adenine modulates the charge densities at the WC site. On going from structure **1** to structure **2**, the charge densities at the WC sites are marginally increased (Figure 2).

Fluorescence and UV–vis spectroscopic techniques were then employed to ascertain the metal ion recognition properties of both **1** and **2** in solution.

**Figure 3:**
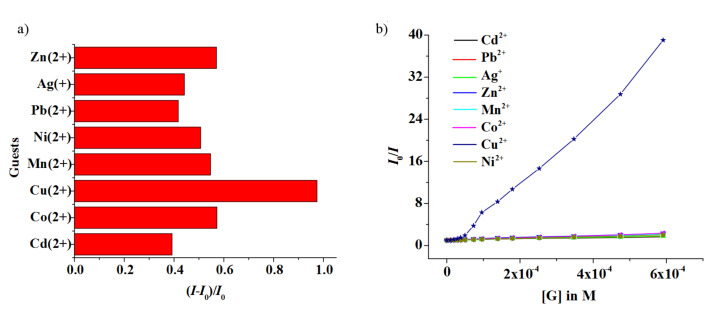
a) Fluorescence ratio (*I*-*I*_0_/*I*_0_) of receptor **1** (*c* = 5.09 × 10^−5^ M) at 413 nm upon addition of 20 equiv of a particular guest in CH_3_CN containing 0.02% DMSO (λ_ex_ = 348 nm); b) Stern–Volmer plot of receptor **1** (*c* = 5.09 × 10^−5^ M) at 413 nm upon addition of 20 equiv of a particular guest in CH_3_CN containing 0.02% DMSO (λ_ex_ = 348 nm).

**Figure 4:**
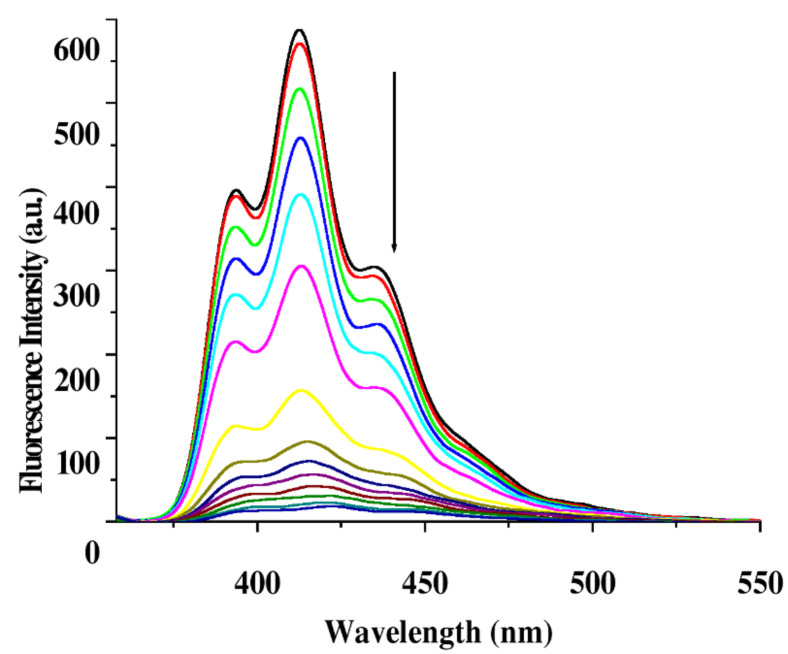
Emission spectra of receptor **1** (*c* = 5.09 × 10^−5^ M) during titration with Cu^2+^ ion (*c* = 1.4 × 10^−3^ M) in CH_3_CN containing 0.02% DMSO (λ_ex_ = 348 nm).

Receptor **1** (*c* = 5.09 × 10^−5^ M) upon excitation at 348 nm in CH_3_CN containing 0.02% DMSO showed structured emission centered on 413 nm. Upon the gradual addition of metal ions as their perchlorate salts to the receptor solution of **1**, the emission of **1** decreased by different extents. Figure 3a demonstrates the change in emission of **1** upon the addition of 20 equivalents of metal ion and clearly shows that the emission of **1** is quenched to the greatest extent by the Cu^2+^ ion. Figure 3b is the Stern–Volmer plot, which gives a comparative view on the quenching of emission of **1**. The almost linear nature of the curves for **1** in Figure 3b corroborates the quenching dynamic in nature and shows a clear distinction between Cu^2+^ ion and the other metal ions investigated. During interaction of **1** with metal ions no additional peak at higher wavelengths either for excimer or exciplex formation should be observed. Figure 4 shows the spectral change of **1** upon titration with Cu^2+^ metal ions.

**Figure 5:**
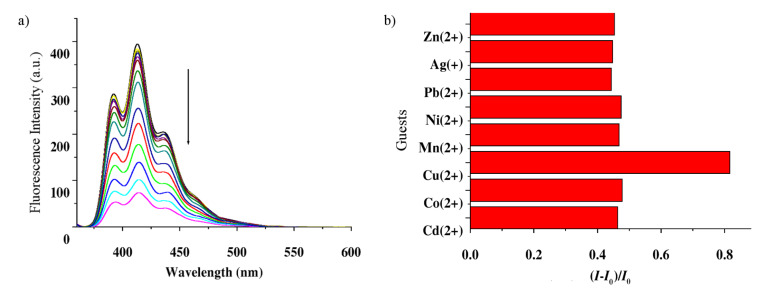
a) Emission spectra of receptor **2** (*c* = 1.48 × 10^−5^ M) during titration with Cu^2+^ ion (*c* = 4.4 × 10^−4^ M) in CH_3_CN containing 0.02% DMSO (λ_ex_ = 348 nm); b) fluorescence ratio (*I*-*I*_0_/*I*_0_) of receptor **2** (*c* = 1.48 × 10^−5^ M) at 413 nm upon addition of 20 equiv of a particular guest in CH_3_CN containing 0.02% DMSO (λ_ex_ = 348 nm).

**Figure 6:**
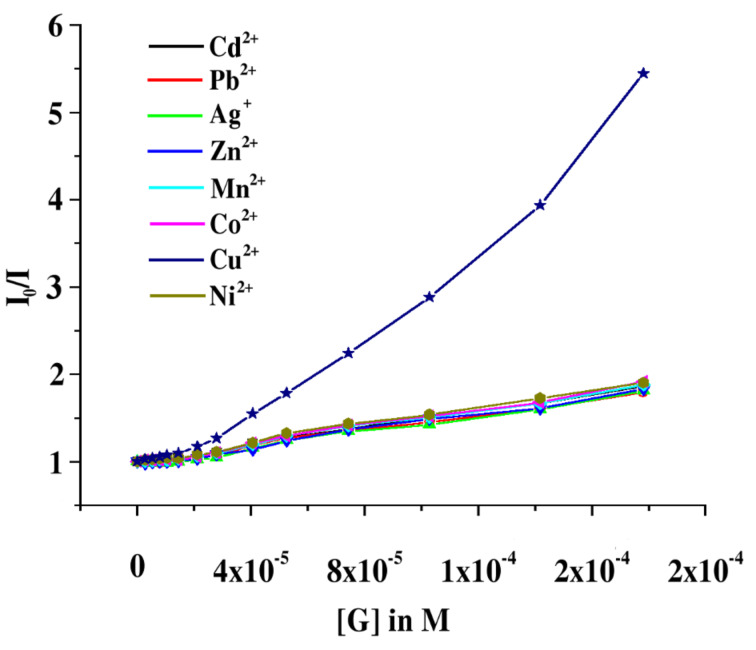
Stern–Volmer plot of receptor **2** (*c* = 1.48 × 10^−5^ M) at 413 nm upon addition of 20 equiv of a particular guest in CH_3_CN containing 0.02% DMSO (λ_ex_ = 348 nm).

A similar trend in emission behavior was observed with receptor **2**. Receptor **2** (*c* = 1.48 × 10^−5^ M) gave an emission spectra of triplet nature with the highest peak at 413 nm on excitation at 348 nm. The emission intensity of **2** was quenched on titration with metal ion solutions as used with compound **1**. Like **1**, the maximum quenching occurred on the successive addition of the Cu^2+^ salt to a solution of receptor **2**. Figure 5a shows the changes in emission intensity of receptor **2** during complexation with Cu^2+^ ions. Titration experiments were also performed with the other metal salts, which also caused quenching, but not to the same extent as with Cu^2+^ ion. Figure 5b shows a comparative view in the change in emission of **2** during titration with different metal ion solutions and Stern–Volmer plot in Figure 6 represents the linear nature of the curves indicating dynamic quenching during the interaction process.

From both Figure 5b and Figure 6 it is evident that receptor **2** also shows a similar fluorometric response as observed for **1** and selectivity for Cu^2+^ ions. The only difference between **1** and **2** is the extent of quenching of emission. The greater degree of quenching of emission in both **1** and **2** in presence of Cu^2+^ ions may presumably occur by the chelation enhanced anthracene → Cu^2+^ π-cation interactions, the paramagnetic effect of Cu^2+^ [34–36].

**Figure 7:**
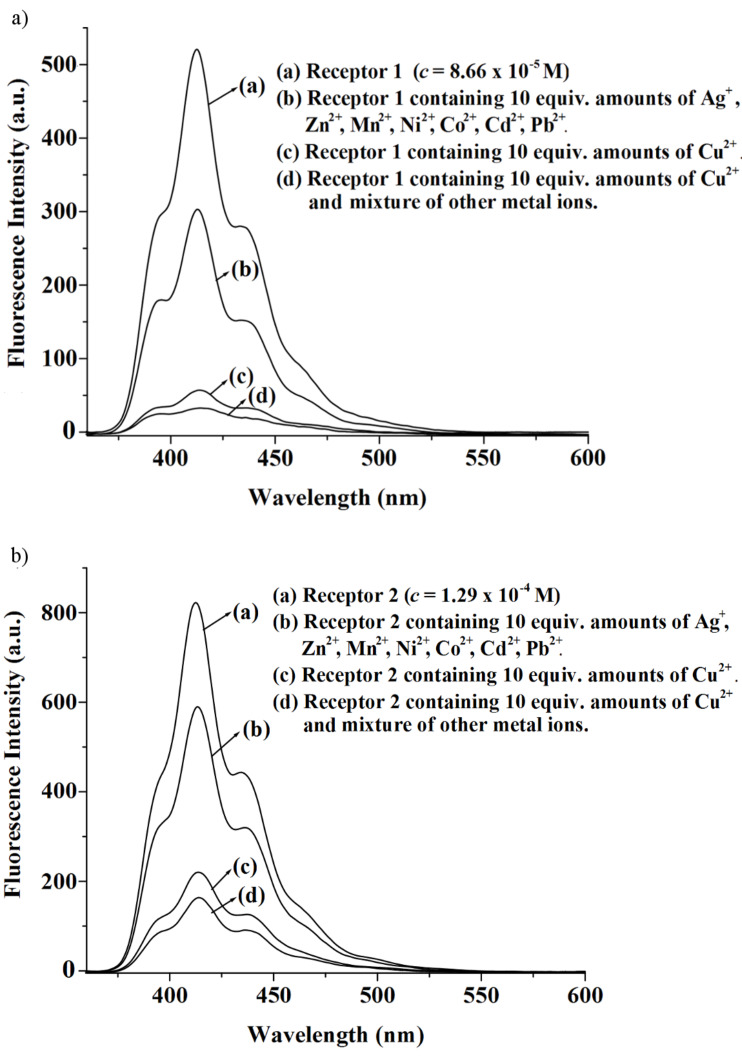
Change in emission of **1** (*c* = 8.66 × 10^−5^ M) (a) and **2** (*c* = 1.29 × 10^−4^ M) (b) upon addition of 10 equiv amounts of Cu^2+^ in the presence and absence of other metal ions in CH_3_CN containing 0.2% DMSO.

To understand the selective sensing of Cu^2+^ by both **1** and **2**, we recorded the emission spectra of the receptors with the addition of 10 equivalents of Cu^2+^ to the receptor solutions containing 10 equivalents of a mixture of other metal ions examined in the present study. Figure 7 displays the comparative view of the change in emission of **1** and **2** in the presence of Cu^2+^ when the other metal ions are absent and present in the receptor solutions. The greater quenching of emission upon addition of Cu^2+^ to the solutions of both **1** and **2** containing other metal ions (Figure 7) corroborates the selectivity in the binding process.

**Figure 8:**
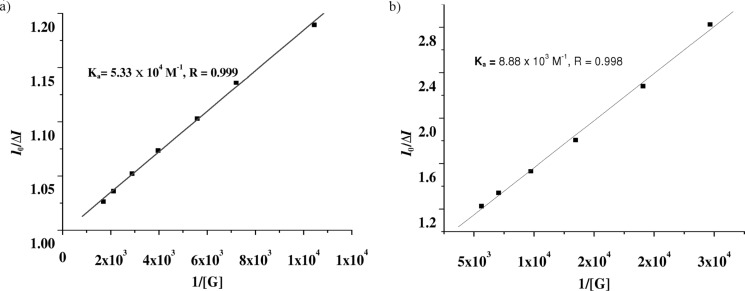
a) Binding constant curve for **1** with Cu^2+^ ion; b) binding constant curve for **2** with Cu^2+^ ion.

The relative change in emission intensity of **1** at 413 nm was used to determine the binding constants [37]. The measured emission (*I*_0_/Δ*I*) at 413 nm when plotted against the inverse of the concentration of guest solution fits almost a linear relationship. The ratio of the intercept versus slope gives the association constants (Table 1). A similar method for determining the binding constants with **2** was employed. As can be seen from Table 1, the receptor **1** has a marked selectivity for Cu^2+^ over other metal ions. A similar feature is noted for **2** but the efficiency in selectivity is found to be less when compared to **1**. This is due to the more basic character of the amino group as well as the ring nitrogen at WC site in **1** in the presence of the anthracenyl group on the amine functionality. The binding constant curves are given in Figure 8 and the linear nature of the curves establishes 1:1 binding model during the interaction process.

**Table 1:** Association constants (*K*_a_ in M^−1^) for **1** and **2** with the metal ions by fluorescence method.

Guests^a^	*K*_a_^b,d^ for **1**	*K*_a_^c,d^ for **2**

Cu^2+^	5.33 × 10^4^	8.88 × 10^3^
Ag^+^	2.17 × 10^2^	1.40 × 10^3^
Co^2+^	8.80 × 10^2^	5.16 × 10^3^
Cd^2+^	2.21 × 10^3^	5.72 × 10^3^
Pb^2+^	1.89 × 10^3^	5.78 × 10^3^
Mn^2+^	9.10 × 10^2^	5.09 × 10^3^
Ni^2+^	2.01 × 10^3^	3.41 × 10^3^
Zn^2+^	3.37 × 10^3^	1.19 × 10^3^

^a^Perchlorate salts were used; ^b^Determined at 413 nm; ^c^Determined at 413 nm; ^d^Errors in *K*_a_ were ≤ 5%.

**Figure 9:**
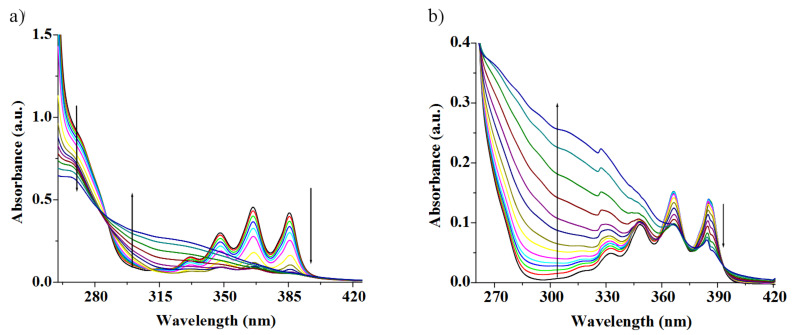
a) Absorption spectra of receptor **1** (*c* = 5.09 × 10^−5^ M) upon gradual addition of Cu^2+^ ion (*c* = 1.4 × 10^−3^ M) in CH_3_CN containing 0.02% DMSO; b) absorption spectra of receptor **2** (*c* = 1.48 × 10^−5^ M) upon gradual addition of Cu^2+^ ion (*c* = 4.4 × 10^−4^ M) in CH_3_CN containing 0.1% DMSO.

**Figure 10:**
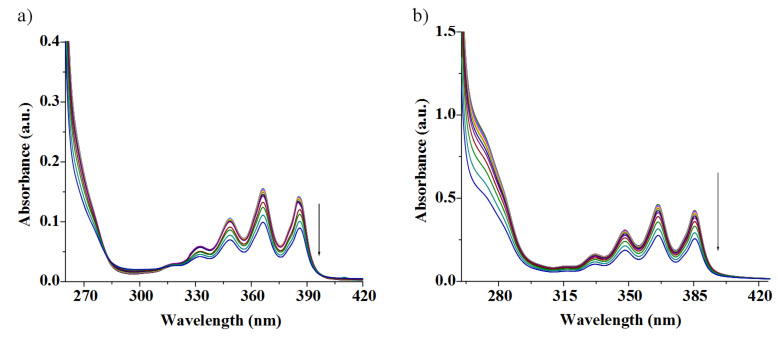
a) Absorption spectra of receptor **2** (*c* = 1.48 × 10^−5^ M) upon gradual addition of Ag^+^ ion (*c* = 4.4 × 10^−4^ M) in CH_3_CN containing 0.1% DMSO; b) absorption spectra of receptor **1** (*c* = 5.09 × 10^−5^ M) upon gradual addition of Ag^+^ ion (*c* = 1.4 × 10^−3^ M) in CH_3_CN containing 0.02% DMSO.

The ground state interaction properties of **1** and **2** were also investigated with the same metal ions in CH_3_CN containing 0.1% DMSO. The concomitant changes in absorption spectra of **1** and **2** were, however, only minor for all metal ions examined with the exception of Cu^2+^. Figure 9a shows the changes in the absorption spectra of **1** upon titration with Cu^2+^ ions. The greater change in absorption of **1** at 268 and 325 nm produced an isosbestic point, indicating the presence of a unique complex in equilibrium with the neutral receptor. The progressive decrease in absorbance at 366 nm for anthracene moiety further suggests the strong participation of anthracene in a π-cation complexation event. Similarly, receptor **2** (*c* = 1.48 × 10^−5^ M) showed intense absorption bands, characteristic of the anthracene moiety at 348, 365 and 385 nm in CH_3_CN containing 0.1% DMSO. Upon adding metal ions as their perchlorate salts the absorption intensity decreased to the different extents. In case of Cu^2+^, the absorption changed significantly in the region at 314 nm and was accompanied by an isosbestic point at 372 nm, which indicates the formation of a supramolecular complex between **2** with Cu^2+^ ion. Figure 9b displays the change in absorption of **2** with addition of Cu^2+^ ions in CH_3_CN containing 0.1% DMSO. It is of note that the change in absorbance of the peaks for anthracene moiety is small compared to that in the case of **1** with Cu^2+^ thereby suggesting a relatively weak cation-π interaction with the anthracenyl function, situated at the distal position from the interacting region. Such findings in the ground state were not observed in case of other cations even with Ag^+^ (Figure 10a for receptor **2**). This was also found for **1** with Ag^+^ ions (Figure 10b). The resulting isotherm fits nicely a 1:1 binding model (Supporting Information File 1).

**Figure 11:**
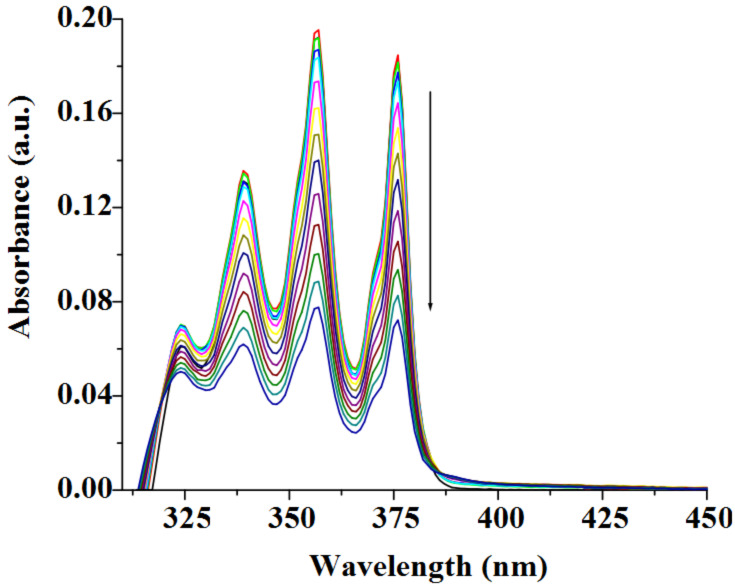
Absorption spectra of anthracene (*c* = 2.39 × 10^−5^ M) upon gradual addition of Cu^2+^ ion (*c* = 1.20 × 10^−3^ M) in CH_3_CN containing 0.1% DMSO.

However, the cation-π interaction in both **1** and **2** is due solely to the presence of adenine moiety which is involved in the binding of metal ions. This was proved by carrying out the UV–vis titration experiment using anthracene only. Figure 11 shows the decrease in absorbance of anthracene upon gradual addition of Cu^2+^ ions. It is of note that the decrease in absorbance of anthracene is much less compared to the cases of **1** and **2** with Cu^2+^. During the interaction process no other changes were observed. This finding thus indicates that the adenine moiety in both **1** and **2** acts as a metal ion binding site for which participation of anthracene in cation-π interaction is facilitated upon binding of metal ions, especially Cu^2+^ ion.

## Conclusion

In conclusion, the above studies have led to the development of adenine–linked fluorescent probes **1** and **2**, which selectively respond to copper ions. The chemosensors display fluorescent changes upon complexation with Cu^2+^ ions. The emission of **1** is greatly decreased in the presence of Cu^2+^ compared to **2** and thus underlines the fact that the sensing ability for metal ions of adenine motif is appreciable and effective when the fluorescent probe resides in close proximity to the WC/HG site. We believe that these observations regarding the interaction between the adenine moiety and Cu^2+^ ions should serve as the basis for new strategies to design new chemosensors based on adenine even although copper ion recognition by other systems has been described. Further progress along this direction is underway in our laboratory.

## Supporting Information

File 1 Stoichiometry curves for **1** and **2** with Cu^2+^ ions.
