# Supplementary material for: Anthracene coupled adenine for the selective sensing of copper ions
Source: Beilstein J Org Chem. 2010 May 5;6:44. doi: 10.3762/bjoc.6.44 (PMC2887277; doi:10.3762/bjoc.6.44)
Supplement: File 1 — Stoichiometry curves for 1 and 2 with Cu2+ ions. [file Beilstein_J_Org_Chem-06-44-s001.pdf]

Supporting Information

for

Anthracene coupled adenine for the selective sensing of copper ions

Kumaresh Ghosh* and Tanushree Sen

Address: Department of Chemistry, University of Kalyani, Kalyani-741235, India.

Email: Kumaresh Ghosh - ghosh_k2003@yahoo.co.in

* Corresponding author

Stoichiometry curves for **1** and **2** with Cu^{2+} ions.

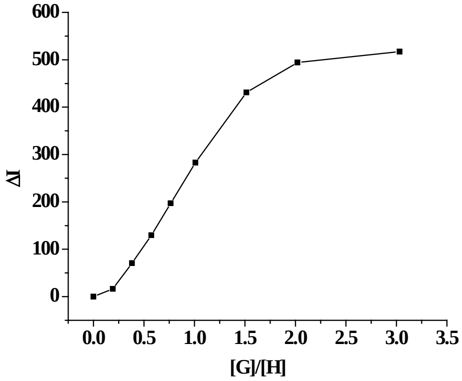

Figure S1: Stoichiometry plot for receptor **1** upon addition of Cu^{2+} ion from fluorescence.

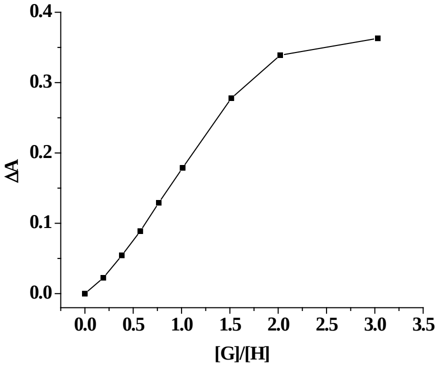

Figure S2: Stoichiometry plot for receptor **1** upon addition of Cu^{2+} ion from UV.

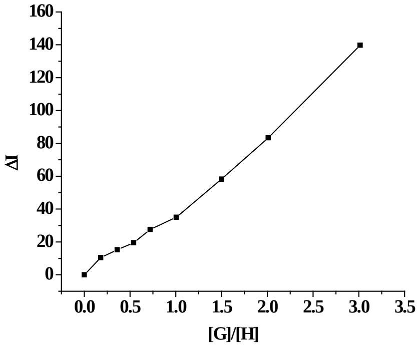

Figure S3: Stoichiometry plot for receptor **2** upon addition of Cu^{2+} ion from fluorescence.
